# COPD Stability and Asthma Remission: Different Words for the Same Therapeutic Ambition?

**DOI:** 10.3390/biomedicines14081675

**Published:** 2026-07-25

**Authors:** Lorenzo Carriera, Pier-Valerio Mari, Roberto Lipsi, Simone Ielo, Eugenio De Corso, Stefano Baglioni, Alberto Ricci, Luca Richeldi

**Affiliations:** 1 Faculty of Medicine and Surgery, Catholic University of Sacred Heart, 00168 Roma, Italy; 2 Pulmonology and Respiratory Intensive Care Unit, Santa Maria della Misericordia Hospital, 06156 Perugia, Italy; roberto.lipsi@ospedale.perugia.it (R.L.); stefano.baglioni@ospedale.perugia.it (S.B.); 3 Internal Medicine, San Carlo di Nancy Hospital, 00165 Rome, Italy; pvmari@gvmnet.it; 4 UOC Pneumologia e UTIP, Ospedale San Donato, USL Toscana SudEst, 52100 Arezzo, Italy; simone.ielo@uslsudest.toscana.it; 5 Section of Otorhinolaryngology, Department of Medicine and Surgery, Santa Maria della Misericordia Hospital, 06156 Perugia, Italy; eugenio.decorso@unipg.it; 6 Division of Pneumology, Department of Clinical and Molecular Medicine, Sapienza University of Rome, AOU Sant’Andrea, 00189 Rome, Italy; alberto.ricci@uniroma1.it; 7 UOC Pneumologia, Dipartimento Neuroscienze, Organi di Senso e Torace, Fondazione Policlinico Universitario A. Gemelli IRCCS, 00168 Rome, Italy; luca.richeldi@policlinicogemelli.it

**Keywords:** COPD stability, asthma remission, chronic obstructive pulmonary disease, type 2 inflammation, treatable traits

## Abstract

The therapeutic goals related to chronic airway diseases are evolving from short-term symptom control toward sustained suppression of disease activity and prevention of future risk. In severe asthma, this shift has been captured by the concept of clinical remission, generally defined by absence of exacerbations, no need for oral corticosteroids, symptom control, and stable or improved lung function. In chronic obstructive pulmonary disease (COPD), the analogous concept has more often been described as disease stability. Although remission in asthma and stability in COPD have developed within different biological and clinical frameworks, they may reflect disease-specific expressions of the same therapeutic ambition. Recent studies support COPD stability as a measurable and clinically meaningful state, associated with reduced exacerbation risk and mortality. Evidence from optimized inhaled triple therapy, particularly with fluticasone furoate/umeclidinium/vilanterol, indicates that multidimensional stability can be achieved and maintained in a proportion of patients, while real-world studies reinforce its applicability beyond randomized trials. The emergence of biologic therapies for selected patients with eosinophilic or type 2 COPD further strengthens the rationale for considering stability as an ambitious treatment target. In this narrative review, informed by a structured literature search, we discuss the conceptual relationship between asthma remission and COPD stability, and summarize the evidence supporting disease stability as an attainable and prognostically relevant outcome. In addition, we propose a pragmatic multidimensional definition based on symptom stability, absence of moderate or severe exacerbations, no systemic corticosteroid use, and maintained lung function over 12 months. COPD stability should not be viewed as a weaker goal than asthma remission, but rather as the most appropriate COPD-specific expression of sustained low disease activity.

## 1. Introduction

The therapeutic goals related to chronic airway diseases are changing. In severe asthma, the concept of clinical remission has progressively entered both scientific discussion and clinical practice. Remission is generally understood as a sustained state of absent or minimal disease activity, characterized by no exacerbations, no need for oral corticosteroids, symptom control, and stable or improved lung function [1,2,3]. In chronic obstructive pulmonary disease (COPD), by contrast, treatment success is more often described as disease stability. At first glance, remission in asthma and stability in COPD may appear to reflect different levels of therapeutic ambition. However, this distinction may be more semantic than conceptual.

Asthma and COPD have traditionally been interpreted through different biological and clinical frameworks. Asthma is usually considered a variable and potentially reversible inflammatory disease, whereas COPD is perceived as progressive, structurally established, and only partially reversible. This difference has shaped therapeutic expectations. In asthma, particularly in patients with type 2 inflammation, biologic therapies can suppress key inflammatory pathways, reduce exacerbations, avoid systemic corticosteroid exposure, improve symptoms, and preserve lung function [1,2,3]. For this reason, remission has become a realistic and increasingly accepted objective. Importantly, remission does not imply cure, but rather a sustained state of very low disease activity. COPD has historically been approached with greater caution. Persistent airflow limitation, emphysema, chronic bronchitis, mucus hypersecretion, cumulative exposure-related injury, and systemic consequences all make complete disease reversal unlikely. For this reason, the term “remission” has rarely been applied to COPD. Yet the emerging concept of disease stability may be closer to asthma remission than is often acknowledged [4]. A stable patient with COPD is not simply a patient whose disease has not worsened. Ideally, stability should mean absence of moderate or severe exacerbations, no need for systemic corticosteroids, improved symptoms and better quality of life, preserved respiratory function and maintained exercise tolerance. These objectives substantially overlap with those used to define remission in asthma.

## 2. Methods

This article is a narrative review informed by a structured literature search. PubMed/MEDLINE, Embase, and Scopus were searched from database inception to 1 July 2026. The principal search combined terms relating to COPD (“chronic obstructive pulmonary disease” OR “COPD”) with terms describing control or stability (“clinical control,” “disease control,” “clinical stability,” “disease stability,” “stable disease,” “low disease activity,” OR “clinically important deterioration”). Additional searches combined these terms with treatment-related keywords, including “triple therapy,” “ICS/LABA/LAMA,” “biologic therapy,” “dupilumab,” and “mepolizumab.” A complementary search combining “asthma” with “clinical remission” or “remission” was conducted to inform the conceptual comparison between the two diseases. Reference lists of eligible articles and relevant reviews were also screened manually to identify additional publications. Eligible publications included English-language conceptual articles, consensus documents, randomized controlled trials, post hoc analyses, prospective and retrospective cohort studies, and real-world studies that proposed, operationalized, validated, or evaluated definitions of COPD clinical control or disease stability. Studies evaluating treatments were considered when they reported composite stability endpoints or outcomes directly relevant to stability, including symptoms or health status, moderate or severe exacerbations, systemic corticosteroid exposure, and lung-function preservation. Conference abstracts were considered only when they reported relevant original data not yet available as a full-text publication. Case reports, studies focused exclusively on the acute management of exacerbations without longitudinal assessment, and publications without a relevant conceptual or operational contribution were excluded. Owing to the conceptual and narrative purpose of the review and the heterogeneity of the included study designs, findings were synthesized descriptively rather than quantitatively. Particular attention was given to the domains included in each definition, the operational thresholds applied, the assessment period, clinical applicability, and associations with subsequent outcomes.

## 3. Asthma Remission as a Therapeutic Framework

Clinical remission in asthma has emerged as a multidimensional treatment objective that extends beyond conventional disease control. Although definitions vary, the most consistently included components are sustained absence of exacerbations, no requirement for systemic corticosteroids, well-controlled or absent symptoms, and stable or improved lung function [1,5]. The recent EUFOREA consensus statement emphasizes the importance of pragmatic and clinically applicable definitions and supports a minimum assessment period of 12 months to ensure that remission reflects a sustained clinical state rather than a transient improvement [6]. Biologic therapies have made clinical remission an increasingly realistic objective in severe asthma by enabling simultaneous improvements across several of these domains [7,8]. Nevertheless, remission is not achieved uniformly, and reported rates vary according to the definition applied, the characteristics of the population, comorbidity burden, treatment duration, and the criteria used for symptoms and lung function. The EUFOREA framework therefore presents remission as an ambitious but achievable treatment target and highlights the importance of individualized, phenotype- and endotype-directed management [6,8]. An important distinction is made between remission on treatment and remission off treatment. Remission on treatment may be achieved while maintenance therapy, including biologic treatment, is continued, whereas remission off treatment requires persistence of the remission state after treatment withdrawal [5,6]. Evidence regarding remission off treatment remains limited, and the optimal duration of therapy, criteria for treatment withdrawal, and likelihood of maintaining remission after discontinuation have not yet been established. Moreover, current definitions remain predominantly clinical, while the potential inclusion of inflammatory or immunological remission, resolution of bronchial hyperresponsiveness, improvement in airway remodeling, and other objective markers remains under discussion [6,8]. Within this framework, asthma remission provides a useful conceptual comparator for COPD stability. Both constructs assess treatment success as a multidimensional and sustained clinical state rather than as improvement in an isolated outcome, although their operational definitions must remain disease-specific because of differences in biology, reversibility, structural burden, and expected disease trajectories.

## 4. Disease Stability in COPD: From Passive Description to Therapeutic Target

The conceptual foundations of COPD control predate the recent disease-stability literature. In 2014, Soler-Cataluña et al. proposed clinical control as the long-term maintenance of a state of low clinical impact adapted to disease severity. This framework combined a cross-sectional dimension—clinical impact, assessed through symptoms, rescue-medication use, physical activity, sputum characteristics, or CAT (COPD Assessment Test)—with a longitudinal dimension of stability, defined principally by the absence of exacerbations and clinical worsening over the preceding 3 months [9,10]. Subsequent prospective studies evaluated and refined these criteria. In the international study by Miravitlles et al., control was defined as the combination of low clinical impact and stability, based on the absence of exacerbations and worsening in CAT or Clinical COPD Questionnaire (CCQ) during the preceding 3 months; the results also indicated that some of the original thresholds were overly restrictive [11]. Modified control criteria were subsequently developed and prospectively validated by Soler-Cataluña et al., with controlled patients showing better health status and a lower risk of subsequent adverse clinical events [12]. Recent literature has strengthened this perspective. Disease stability has been proposed as an appropriate and attainable COPD treatment goal, building on these existing concepts of clinical control and clinically important deterioration, but moving beyond short-term symptom improvement alone [4]. In this framework, stability is not a passive description of non-worsening disease, but a measurable therapeutic target that integrates clinical, functional, and prognostic dimensions [4]. This is clinically important because COPD management should not be limited to reacting to deterioration after it occurs; it should aim to preserve the patient’s best achievable state over time. Loo et al. [13] provided one of the first prospective longitudinal evaluations of clinical stability in COPD. In their study, clinical stability was defined pragmatically as the absence of exacerbations together with stable symptoms, assessed by a CAT change in less than 2 points from baseline over a 2-year period. Approximately one third of patients achieved clinical stability at 2 years, supporting the idea that stability is attainable in a relevant proportion of patients with COPD. Importantly, the study also identified several baseline factors associated with a higher probability of achieving 2-year stability, including higher body mass index, better post-bronchodilator FEV_1_/FVC ratio, fewer baseline exacerbations, absence of bronchiectasis, preserved haemoglobin levels, and smoking cessation. However, Loo et al. [13] also showed that clinical stability at 2 years did not independently predict subsequent mortality, whereas cardiovascular disease was associated with 5-year mortality risk. The prognostic relevance of COPD stability has recently been supported by cohort evidence. Kim et al. [14] defined disease stability over 1 year as the simultaneous presence of symptom stability, absence of moderate or severe exacerbations, and absence of rapid lung-function decline. In this retrospective cohort, 39% of patients achieved stability, which was associated with a lower adjusted risk of subsequent moderate-to-severe exacerbations, severe exacerbations, and all-cause mortality during long-term follow-up. Similarly, Son et al. [15] externally validated the clinical relevance of disease stability in the Korea COPD Subgroup Study cohort, showing that patients with stable disease experienced fewer exacerbations and lower mortality. Together, these studies support COPD stability as a reproducible and prognostically meaningful clinical state, rather than a simple description of absent short-term deterioration.

## 5. Evidence from Optimized Inhaled Triple Therapy

Optimized inhaled therapy provides the clearest evidence that stability is an attainable goal in COPD. Triple therapy with an inhaled corticosteroid, a long-acting beta-agonist, and a long-acting muscarinic antagonist (ICS/LABA/LAMA) has demonstrated the ability to reduce exacerbations, improve symptoms and health status, and help preserve lung function. In FULFIL, fluticasone furoate/umeclidinium/vilanterol (FF/UMEC/VI) improved lung function and health-related quality of life compared with budesonide/formoterol [16]. In a post hoc analysis of this study, COPD stability was defined after 24 weeks using a composite endpoint comprising no moderate or severe exacerbations, an FEV_1_ decline ≤100 mL from baseline, and no clinically meaningful deterioration in health status, defined as SGRQ worsening ≤4 points or CAT worsening ≤2 points. Patients treated with FF/UMEC/VI maintained this stable state for a substantially longer period than those receiving budesonide/formoterol, and maintained this state significantly longer [17]. These findings were further supported by IMPACT, in which FF/UMEC/VI reduced moderate or severe exacerbations compared with FF/VI and UMEC/VI, confirming its role in future-risk reduction [18], and by its post hoc analysis [19], which showed that a greater proportion of patients receiving FF/UMEC/VI achieved disease stability at week 52 and maintained it for longer than those receiving dual therapy [19]. These findings were subsequently extended by Singh et al. through post hoc analyses of the IMPACT and FULFIL trials using a common three-component definition of disease stability, comprising no moderate or severe exacerbations and no worsening from baseline in CAT score or FEV_1_. Across both trials, FF/UMEC/VI increased the likelihood of achieving stability compared with dual therapy. Stability was achieved by 22% at Week 52 in IMPACT and by 46% at Week 24 in FULFIL. Importantly, achievement of stability at Week 28 in IMPACT was associated with a 45.7% lower subsequent risk of moderate or severe exacerbations and a 51.7% lower risk of all-cause mortality [20]. Similarly, real-world data showed that clinical stability after 12 months of FF/UMEC/VI treatment, defined by absence of exacerbations, CAT improvement, and limited FEV_1_ decline, can be achieved in routine clinical practice [21]. This observation is further supported by a 12-month retrospective cohort of exacerbating patients with COPD treated with once-daily FF/UMEC/VI, in which 73% achieved a composite disease-stability endpoint based on absence of moderate or severe exacerbations, no clinically meaningful CAT worsening, and no significant FEV_1_ decline [22]. Treatment was also associated with improved lung function, reduced hyperinflation, better CAT scores, and fewer moderate or severe exacerbations, reinforcing the relevance of multidimensional stability assessment beyond randomized clinical trials [22].

Although composite stability endpoints have been more extensively evaluated with FF/UMEC/VI, evidence from other single-inhaler triple therapies is broadly consistent with the same concept of improved long-term disease control and risk reduction. Randomized trials and real-world studies with budesonide/glycopyrronium/formoterol (BGF) and extrafine beclometasone dipropionate/formoterol/glycopyrronium (BDP/FF/G) have shown benefits on exacerbations, lung function, symptoms and health status [23,24,25,26,27,28]. In the prospective RESPONSE study, disease stability was defined as the simultaneous absence of exacerbations, a CAT increase <2 points, and an FEV_1_ decline <100 mL over 26 weeks; 53% of patients achieved this composite endpoint after switching from dual therapy to extrafine BDP/FF/G, alongside improvements in symptoms, lung function, quality of life, and treatment adherence [29]. These data support optimized ICS/LABA/LAMA therapy as a class-level foundation for pursuing COPD stability, while highlighting the need to apply standardized composite stability definitions across different triple-therapy regimens.

Published frameworks have operationalized COPD clinical control and disease stability using partially overlapping but non-identical constructs (Table 1). Earlier definitions of clinical control combined a cross-sectional assessment of disease impact with short-term stability, generally evaluated over approximately 3 months. More recent disease-stability studies have instead used multidimensional composite endpoints incorporating exacerbations, symptoms or health status, and lung-function trajectory over periods ranging from 24 weeks to 2 years. Symptom stability has been defined as either no worsening or clinically meaningful improvement, whereas lung-function preservation has ranged from no decline to an annual FEV_1_ decline below 60 or 100 mL. These differences currently limit comparisons across studies and underline the need for a simple and standardized operational definition.

## 6. Biologic Therapy and the Expansion of Stability in Eosinophilic/Type 2 COPD

The recent introduction of biologic therapy for selected patients with eosinophilic or type 2 COPD makes this discussion even more relevant.

Dupilumab, added to optimized inhaled triple therapy, has reduced moderate or severe exacerbations and improved lung function, health-related quality of life, and respiratory symptoms in patients with COPD and type 2 inflammation [30,31].

The inhibition of the IL-5 pathway has also strengthened the role of mechanism-based treatment in eosinophilic COPD. In MATINEE, mepolizumab reduced the annualized rate of moderate or severe exacerbations by 21% in patients with blood eosinophils ≥300 cells/µL and persistent exacerbation risk despite optimized inhaled therapy, confirming and extending earlier signals from METREX and METREO [32,33]. By targeting eosinophil-driven inflammation and reducing exacerbation susceptibility, mepolizumab approval reinforces the idea that COPD stability may be achieved through biologically guided treatment in carefully selected phenotypes [34].

At present, however, direct evidence that biologic therapies induce a multidimensional state of COPD stability remains limited. The pivotal dupilumab and mepolizumab trials were designed primarily to evaluate exacerbation rates and other individual efficacy outcomes rather than stability as a prespecified composite endpoint [30,31,32,33]. Accordingly, the available evidence remains domain-specific rather than composite and does not establish that the same patients simultaneously achieved symptom stability, exacerbation freedom, preserved lung function, and avoidance of systemic corticosteroid exposure.

More direct evidence has recently emerged from the post hoc analysis by Singh et al., who applied a common three-component definition of stability—no moderate or severe exacerbations and no worsening from baseline in CAT score or FEV_1_—to pooled MATINEE, METREX, and METREO data. At Week 52, stability was achieved by 18% of patients receiving mepolizumab in addition to triple therapy, and the probability of achieving stability was greater with mepolizumab than with placebo [20]. This analysis provides preliminary evidence that biologic treatment may increase the proportion of patients who simultaneously achieve stability across multiple domains. Nevertheless, the analysis was post hoc, was restricted to selected patients with eosinophilic COPD, and evaluated stability at a specific time point rather than its duration. It therefore does not establish whether biologic-induced stability can be maintained over successive years or after treatment discontinuation. The corresponding multidimensional stability outcome has also not yet been prospectively evaluated with dupilumab, despite its demonstrated benefits across several individual stability domains.

In this context, eosinophilic COPD should be distinguished from asthma–COPD overlap (ACO), historically termed ACOS. ACO describes patients with persistent airflow limitation and clinical features of both asthma and COPD, but lacks universally accepted diagnostic criteria and represents a heterogeneous clinical construct rather than a single disease entity [35,36]. Eosinophilic COPD, by contrast, is an inflammatory phenotype or treatable trait characterized by eosinophilic or type 2 inflammation and may occur without a current or previous diagnosis of asthma. Although the two populations may overlap, eosinophilia alone does not identify ACO, and patients with asthma–COPD coexistence may differ clinically and biologically from those with eosinophilic COPD without asthma [37]. This distinction is relevant when interpreting biologic evidence, which remains limited in ACO and should not be directly extrapolated between these populations [38].

Biologic therapies may also influence treatable traits that contribute to recurrent instability. Mucus plugging, for example, is associated with type 2 inflammation and may represent a shared feature across the asthma–COPD spectrum and a potential target for type 2 cytokine blockade [39].

Thus, biologic therapies provide a strong biological and clinical rationale for extending the stability concept to selected eosinophilic or type 2 COPD populations, but the current evidence remains preliminary. Their capacity to induce stability, increase its duration, prevent loss of stability, and reduce long-term disease progression remains an important knowledge gap. Prospective studies specifically designed around standardized multidimensional stability endpoints are required before biologic therapies can be considered established stability-inducing or stability-maintaining treatments.

## 7. Toward a Pragmatic Definition of COPD Stability

Despite growing interest in COPD stability, available definitions remain heterogeneous and are often study-specific. Some definitions emphasize symptom stability and absence of exacerbations, whereas others also include lung-function preservation or health-status improvement. This heterogeneity reflects the complexity of COPD, but it also limits comparison across studies and prevents stability from being used as a standardized endpoint. A pragmatic definition should therefore be simple enough for clinical use, but sufficiently multidimensional to capture the major domains of disease activity, future risk, treatment burden, and functional preservation.

**Figure 1:**
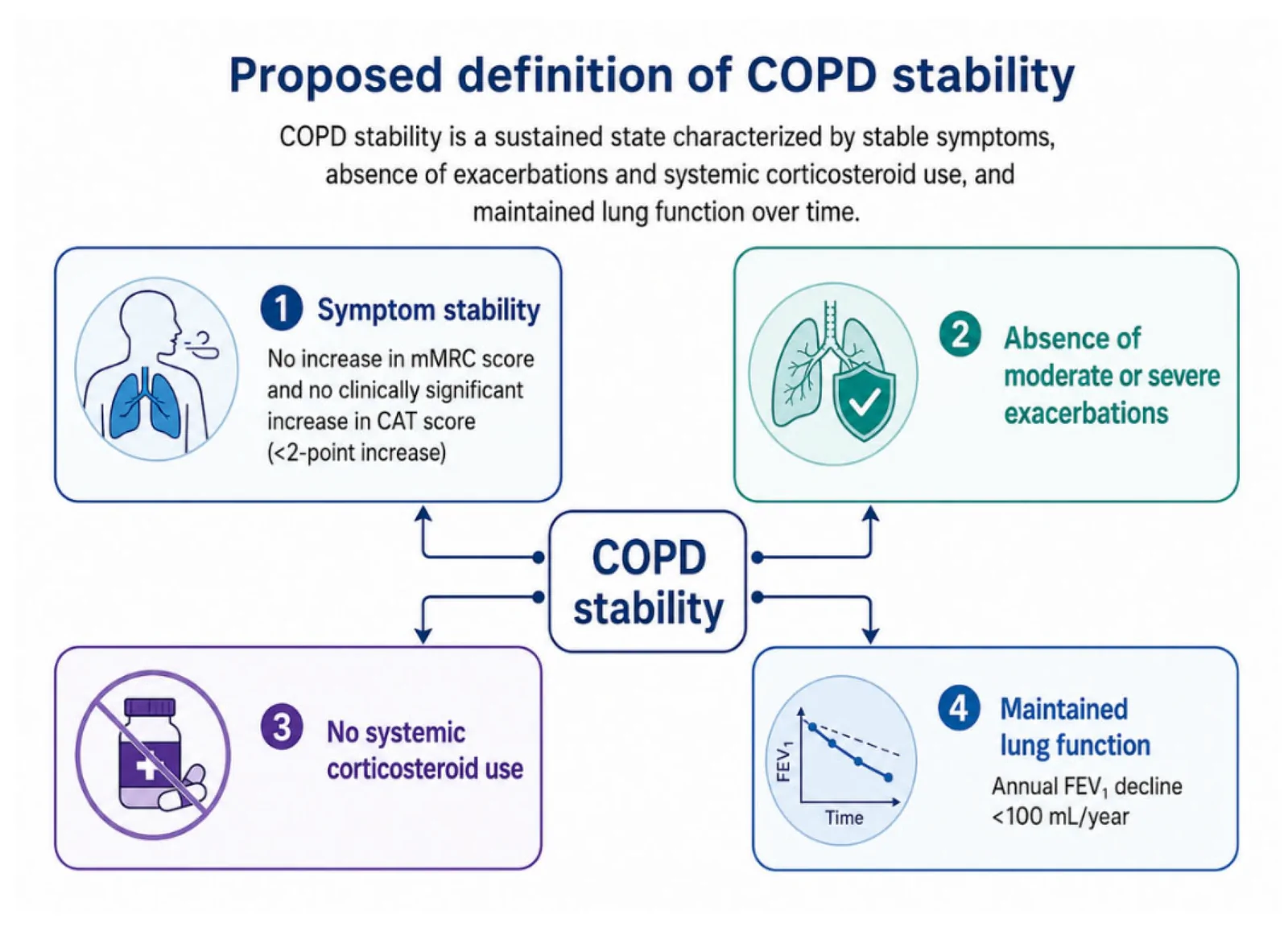
Proposed multidimensional definition of COPD stability.

Based on this framework, we propose a pragmatic multidimensional definition of COPD stability. COPD stability should be considered a sustained state in which four conditions are simultaneously met over 12 months: symptom stability, defined by no increase in mMRC score and no clinically significant worsening in CAT score, defined as <2-point increase; absence of moderate or severe exacerbations; no use of systemic corticosteroids; and maintained lung function, defined as an annual FEV_1_ decline of less than 100 mL/year (Figure 1).

COPD stability is defined by four simultaneous components: symptom stability, absence of moderate or severe exacerbations, no systemic corticosteroid use, and maintained lung function over time. Symptom stability is based on mMRC and CAT, while maintained lung function is defined as an annual FEV_1_ decline <100 mL/year.

Each component was selected to capture a distinct and clinically relevant dimension of COPD activity. Despite the heterogeneity summarized in Table 1, three domains recur across most published definitions: absence of moderate or severe exacerbations, stability of symptoms or health status, and preservation of lung function. These recurring domains therefore constitute the foundation of our proposal. Symptom stability reflects the patient’s daily experience and was defined using CAT and mMRC, two brief and widely used instruments that assess complementary aspects of disease burden. CAT provides a multidimensional assessment of COPD-related health status, whereas mMRC primarily captures dyspnea-related functional limitation. Because a 2-point change represents the most reliable estimate of the minimum clinically important difference for CAT, an increase of <2 points was selected to exclude clinically meaningful worsening while allowing for minor variations that may reflect day-to-day or measurement variability [40]. As mMRC is an ordinal scale, no increase was permitted, thereby avoiding classification of a transition to a worse dyspnea category as stability. Absence of moderate or severe exacerbations was included because these events represent clinically relevant episodes of disease instability, adversely affect subsequent disease trajectory, and are consistently incorporated into previous stability and clinically important deterioration frameworks. Complete freedom from moderate or severe exacerbations was preferred to a reduction in their frequency because the proposed construct is intended to identify a sustained state of low disease activity rather than merely a relative treatment response. Maintained lung function was included as an objective measure of functional disease trajectory. An annual FEV_1_ decline <100 mL was selected because a decrease of ≥100 mL is commonly used as a threshold for clinically important deterioration and has been adopted in several previous definitions of COPD stability [4,14,17,19,20,21,22,29,41]. This threshold also allows for smaller changes that may result from biological variability, measurement variability, or expected age-related decline rather than unequivocal disease progression. FEV_1_ declines below 100 mL over 12 months may still be clinically important, particularly when values fall within the 60–99 mL range. Such changes may indicate an accelerated functional trajectory and should not be interpreted as necessarily physiological, benign, or fully compatible with long-term lung-function preservation. Several longitudinal studies have used thresholds of approximately 60–75 mL/year to identify rapid decliners, although definitions remain heterogeneous and also include distribution-based approaches, such as classification according to the fastest quartile of decline within a study population [42]. Observed FEV_1_ trajectories also vary considerably according to the population examined, baseline disease severity, and duration and frequency of follow-up. For example, markedly different one-year FEV_1_ changes have been reported across mild COPD and high-risk populations, underscoring the heterogeneity of functional decline [43]. Moreover, the rate of FEV_1_ decline is a longitudinal biomarker that is more reliably characterized through repeated measurements over time than through a comparison between only two annual examinations [44].

Absence of COPD-related systemic corticosteroid use was retained as an explicit fourth component. Although systemic corticosteroid-treated exacerbations partly overlap with the exacerbation domain, separate assessment of corticosteroid exposure may additionally capture maintenance treatment and steroid-treated episodes that are not consistently documented or classified as moderate exacerbations. It also provides a direct measure of treatment burden and facilitates conceptual alignment with clinical remission in asthma, in which avoidance of systemic corticosteroids is a central criterion [1,2,3]. Systemic corticosteroid use for conditions unrelated to COPD should not, however, be interpreted as evidence of COPD instability. A 12-month assessment period was selected because it is sufficiently long to account for seasonal variation in symptoms and exacerbations, distinguish sustained stability from a transient treatment response, and remain feasible for routine annual reassessment [4,13,14,15,21,22]. Compared with previously proposed definitions, which vary in their included domains, thresholds, and assessment periods, our proposal requires the simultaneous fulfilment of four complementary criteria over a fixed interval and relies exclusively on measures routinely available in clinical practice. Its intended added value therefore lies in operational clarity, simplicity, multidimensional assessment, and the explicit inclusion of systemic corticosteroid burden. By translating into COPD the same multidimensional logic that underlies clinical remission in asthma, this proposal presents stability as a disease-specific expression of sustained low disease activity.

To facilitate clinical implementation, this multidimensional definition may be translated into a simple stepwise workflow for routine follow-up. Rather than functioning only as a research endpoint, COPD stability could be used as a structured clinical target, prompting clinicians to evaluate symptoms, exacerbations, COPD-related systemic corticosteroid exposure, lung-function trajectory, and modifiable treatable traits during longitudinal assessment [34,45]. These elements are incorporated into the proposed practical workflow shown in Table 2.

This approach would allow stability to function not only as an outcome, but also as a clinical decision tool. Patients who meet stability criteria may continue current treatment with reinforcement of adherence, inhaler technique, smoking cessation, vaccination, pulmonary rehabilitation, and comorbidity management. Conversely, failure to achieve stability should prompt systematic reassessment of modifiable factors and consideration of phenotype-directed treatment optimization.

## 8. Future Directions for COPD Stability

Future studies should validate and refine pragmatic definitions of COPD stability and clarify how this target should be measured, achieved, and sustained over time across different therapeutic contexts, from optimized inhaled therapy to biologic treatment. Before stability can be adopted as a formal therapeutic target, its feasibility, measurement properties, incremental value over existing concepts such as clinical control and CID, and association with long-term outcomes require prospective validation.

At present, available studies have used partly heterogeneous definitions, combining symptom control, exacerbation prevention, health-status preservation, and lung-function stability in different ways. Although this heterogeneity reflects the multidimensional nature of COPD, it limits comparability across studies and prevents the systematic use of stability as a standardized clinical endpoint. A shared operational definition, based on simple and reproducible criteria, is therefore essential to translate the concept of stability from research into routine clinical practice.

Future work should also define the optimal time frame required to assess stability. Shorter intervals, such as 24 or 52 weeks, may be useful to evaluate early treatment response, whereas longer observation periods may be necessary to determine whether stability is sustained and prognostically meaningful. Stability is unlikely to have a uniform meaning across all patients with COPD. Its attainability may depend on phenotype, baseline severity, exacerbation history, comorbidity burden, and treatable traits such as chronic bronchitis, mucus hypersecretion, eosinophilic inflammation, bronchiectasis, cardiovascular disease, or frailty.

Future research should therefore determine whether stability definitions need to be adapted to specific clinical phenotypes, while preserving a common core of outcomes. This may be particularly relevant in eosinophilic or type 2 COPD, where biologic treatment may expand the possibility of achieving sustained low disease activity beyond optimized inhaled therapy alone. Biologic therapy should represent a specific priority for future stability research. Trials should prospectively include multidimensional stability as a prespecified endpoint and determine not only whether stability is achieved, but also how rapidly it occurs, how long it is maintained, and which clinical or biological characteristics predict its attainment. Repeated assessments are needed to distinguish transient response from durable stability and to determine whether loss of stability is preceded by changes in symptoms, biomarkers, lung function, or treatable traits. Future studies should also examine whether biologic-associated stability reduces systemic corticosteroid exposure, hospitalization, mortality, and longer-term functional decline, and whether it persists after treatment discontinuation. Direct comparisons using a shared stability definition will be essential to establish whether different biologic pathways produce comparable or distinct patterns of disease stabilization.

Importantly, stability should not be interpreted as disease absence or cure, nor as a complete substitute for global prognostic assessment. Rather, it should be integrated with broader evaluation of comorbidities, physical activity, hyperinflation, frailty, and patient-centered outcomes. The future role of stability within COPD management will ultimately depend on whether it can reliably identify sustained low disease activity, discriminate between meaningful treatment responses and short-term fluctuations, predict subsequent outcomes, and guide clinical reassessment. Only after these properties have been established should COPD stability be considered for incorporation into the broader therapeutic goals of COPD management.

## 9. Conclusions

In conclusion, stability in COPD should not be considered a weaker or less ambitious goal than remission in asthma. Rather, it may represent the most appropriate COPD-specific expression of the same therapeutic ambition: sustained suppression of disease activity, prevention of future risk, reduction in treatment burden, and preservation of respiratory function and quality of life.

The terminology differs because asthma and COPD differ in biology, reversibility, and structural burden, but the underlying clinical objective is increasingly similar: maintaining the patient in a durable state of low disease activity.

This perspective is particularly relevant in the current therapeutic era. Optimized inhaled triple therapy has already shown that multidimensional stability, including fewer exacerbations, improved symptoms, better health status, and preserved lung function, is attainable in a proportion of patients with COPD.

More recently, biologic therapies targeting type 2 inflammation have further expanded this possibility in selected eosinophilic COPD populations. These advances challenge the traditional view of COPD as a disease in which treatment can only slow decline, and support a more proactive model of care focused on preserving the best achievable clinical state over time.

As treatment options expand, the central question is no longer only how to prevent COPD worsening. It is whether sustained stability can become a measurable and ambitious goal capable of reshaping COPD care. Establishing this concept would provide clinicians with a clearer framework to evaluate treatment success and would help move COPD management from episodic disease control toward sustained preservation of health.

## Figures and Tables

**Table 1:** Definitions, assessment periods, and outcomes used in key studies of COPD clinical control and disease stability.

Study	Study Design or Framework	Operational Definition of Control or Stability	Assessment Period	Outcomes Evaluated
Soler-Cataluña et al., 2014 [9,10]	Conceptual framework and narrative review	Clinical control defined as low clinical impact adapted to disease severity together with stability. Clinical impact included dyspnea, rescue-medication use, physical activity, sputum color, or CAT; stability required absence of exacerbations and clinical worsening.	Previous 3 months	Development and clinical interpretation of a dynamic COPD-control framework intended to support longitudinal assessment and treatment adjustment
Miravitlles et al., 2018 [11]	Prospective multicentre observational study	Control defined as low clinical impact plus stability, with stability based on absence of exacerbations and no worsening in CAT or CCQ.	Previous 3 months	Prevalence and clinical characteristics of controlled COPD; evaluation and sensitivity analysis of the original thresholds
Soler-Cataluña et al., 2018 [12]	Prospective, multicentre observational validation study	Modified control criteria combining low clinical impact with absence of exacerbations and clinical worsening; impact thresholds were adjusted according to BODEx or FEV_1_ severity	Stability assessed over 3 months; clinical outcomes evaluated over 12 months	Achievement of control; health status; time to exacerbation, emergency visit, hospitalization, death, and composite adverse events
Singh et al., 2025 [4]	Narrative review and preliminary conceptual framework	Proposed that disease stability should integrate exacerbation prevention, stable symptoms or health status, and preservation of lung function. MCID-based thresholds derived from CID were discussed, but no definitive validated composite was established.	Suggested longitudinal assessment, generally over 6–12 months	Conceptual development, candidate domains, measurement thresholds, and clinical implementation
Loo et al., 2025 [13]	Prospective longitudinal cohort	No exacerbations requiring OCS, antibiotics, or hospitalization, together with stable symptoms, defined as a CAT change <2 points from baseline	1 and 2 years	Proportion achieving stability, baseline predictors of 2-year stability, and subsequent 5-year mortality
Wang et al., 2025 [17]	Post hoc analysis of FULFIL	No moderate or severe exacerbations, FEV_1_ decline ≤100 mL from baseline, and either SGRQ worsening ≤4 points or CAT worsening ≤2 points	24 weeks	Achievement and duration of stability with FF/UMEC/VI versus budesonide/formoterol, stratified by chronic mucus hypersecretion
Han et al., 2025 [19]	Post hoc analysis of IMPACT	No moderate or severe exacerbations, FEV_1_ decline ≤100 mL from baseline, and either SGRQ worsening ≤4 points or CAT worsening ≤2 points	52 weeks	Proportion achieving stability and duration of stability with FF/UMEC/VI versus FF/VI or UMEC/VI
Kim et al., 2026 [14]	Retrospective cohort	No increase in mMRC score, CAT increase <2 points, no moderate or severe exacerbations, and annual FEV_1_ decline <60 mL/year	1 year	Subsequent moderate or severe exacerbations, severe exacerbations, and all-cause mortality
Son et al., 2026 [15]	Prospective cohort; external validation in KOCOSS	No moderate or severe exacerbations, no decline in FEV_1_, and no increase in SGRQ score	1 year	Exacerbations during the following year, subsequent lung-function decline, and long-term all-cause mortality
Singh et al., 2026 [20]	Post hoc analyses of Phase 3 trials: IMPACT, FULFIL, and pooled MATINEE/METREX/METREO	Three-component composite comprising no moderate or severe exacerbations and no worsening from baseline in CAT score and trough FEV_1_. SGRQ was evaluated as an alternative health-status measure in sensitivity analyses.	IMPACT: Weeks 28 and 52; FULFIL: Week 24; pooled MATINEE/METREX/METREO: Week 52	Proportion achieving stability; treatment comparisons between FF/UMEC/VI and dual therapy and between mepolizumab and placebo added to triple therapy; association of stability with subsequent moderate or severe exacerbations and all-cause mortality
Maniscalco et al., 2026 [21]	Real-world retrospective observational study	No acute exacerbations during the previous 12 months, CAT improvement ≥2 points from baseline, and FEV_1_ decline <100 mL	12 months	Prevalence of stability and changes in symptoms, exacerbations, and lung function after FF/UMEC/VI
Lupia et al., 2026 [22]	Real-world retrospective observational study	No moderate or severe exacerbations, CAT increase ≤2 points, and no FEV_1_ reduction ≥100 mL from baseline	12 months	Achievement of stability, exacerbations, CAT, lung function, hyperinflation, gas transfer, and predictors of stability
Dulai et al., 2026 [29]	Prospective, non-interventional, multicentre real-world study with BDP/FF/G	No exacerbations, no FEV_1_ decrease ≥100 mL, and no CAT worsening ≥2 points	26 weeks	Proportion achieving stability; changes in CAT, FEV_1_, quality of life, symptoms, and treatment adherence

Abbreviations: BDP/FF/G, beclometasone dipropionate/formoterol/glycopyrronium; BODEx, body mass index, airflow obstruction, dyspnea, and exacerbations index; CAT, COPD Assessment Test; CCQ, Clinical COPD Questionnaire; CID, clinically important deterioration; COPD, chronic obstructive pulmonary disease; FEV_1_, forced expiratory volume in 1 s; FF, fluticasone furoate; KOCOSS, Korea COPD Subgroup Study; MCID, minimum clinically important difference; mMRC, modified Medical Research Council dyspnea scale; OCS, oral corticosteroids; SGRQ, St George’s Respiratory Questionnaire; UMEC, umeclidinium; VI, vilanterol.

**Table 2:** Practical workflow for assessing COPD stability in routine clinical follow-up.

Step	Clinical Question	Practical Assessment	Possible Implication
1	Is the patient symptomatically stable?	CAT, mMRC, and health-status or quality-of-life assessment	If unstable, reassess treatment response, inhaler technique, adherence, comorbidities, and activity limitation.
2	Has the patient remained free from exacerbations?	Moderate or severe exacerbations over the previous 12 months	If exacerbations occurred, evaluate exacerbation phenotype, future risk, eosinophilic inflammation, infection, bronchiectasis, and chronic bronchitis.
3	Has systemic corticosteroid use been avoided?	OCS bursts, maintenance OCS, or steroid-treated instability events	If systemic corticosteroids were required, consider uncontrolled disease, recurrent instability, or insufficient control of treatable traits.
4	Is lung function preserved?	Serial spirometry and FEV_1_ trajectory over time	If lung function declines, reassess disease progression, hyperinflation, smoking status, adherence, comorbidities, and the need for treatment optimization. An FEV_1_ decline of 60–99 mL over 12 months should prompt repeat standardized post-bronchodilator spirometry, evaluation of potentially modifiable contributors, and closer monitoring of the longer-term FEV_1_ trajectory.
5	Are modifiable treatable traits present?	Blood eosinophils (preferably assessed longitudinally), mucus hypersecretion, chronic bronchitis, bronchiectasis, smoking, hyperinflation, cardiovascular disease, frailty, and physical inactivity	Personalize pharmacological escalation, biologic eligibility assessment, pulmonary rehabilitation, smoking cessation, vaccination, or comorbidity management.

## Data Availability

No new data were created or analyzed in this study. Data sharing is not applicable to this article.
